# Tear-Film Stability and Ocular Symptoms During Two Years of Low-Dose Atropine Treatment in Children: A Secondary Analysis of the ASPECT Trial

**DOI:** 10.3390/children13101376

**Published:** 2026-10-10

**Authors:** Beatriz Martín-García, Elena Montolío-Marzo, Noemi Guemes-Villahoz, Blanca Alonso-Abad, Alicia Ruiz-Pomeda, Paula Talavero-González, Elena Hernández-García, Rafael Bella-Gala, Nunila Gómez de Liaño, Joel Simbaña-Fierro, Julián García-Feijóo, Rosario Gómez-de-Liaño

**Affiliations:** 1 Department of Optometry and Vision, Faculty of Optics and Optometry, Complutense University of Madrid, 28037 Madrid, Spain; bmarting@ucm.es (B.M.-G.); eeccmiopia.oft.hcsc@salud.madrid.org (B.A.-A.); alicru04@ucm.es (A.R.-P.); rbella@ucm.es (R.B.-G.); mdgomezd@ucm.es (N.G.d.L.); 2 Department of Ophthalmology, Hospital Clínico San Carlos, 28040 Madrid, Spain; elena.montolio@salud.madrid.org (E.M.-M.); paula.talavero@salud.madrid.org (P.T.-G.); ehernandezg@salud.madrid.org (E.H.-G.); jgfeijoo@salud.madrid.com (J.G.-F.); lianooft@ucm.es (R.G.-d.-L.); 3 Health Research Institute of Hospital Clínico San Carlos (IdISSC), Biomedical Research Foundation of Hospital Clínico San Carlos (FIBHCSC), 28040 Madrid, Spain; 4 Ramón Castroviejo Institute for Ophthalmic Research (IIORC), Department of Immunology, Ophthalmology and Otorhinolaryngology, Faculty of Medicine, Complutense University of Madrid, 28040 Madrid, Spain; 5 Research Methodological Support Unit of Preventive Department, Instituto de Investigación Sanitaria del Hospital Clínico San Carlos (IdISSC), 28040 Madrid, Spain; davidjoel.simbana.externo@salud.madrid.org

**Keywords:** childhood myopia, low-concentration atropine, tear-film stability, ocular symptoms, OSDI, fNIBUT, tolerability, compounded formulation

## Abstract

**Highlights:**

**What are the main findings?**

- Over 24 months of nightly compounded 0.025% atropine, no deterioration was detected in tear-film stability or corneal endothelial cell density among 101 children completing follow-up.
- OSDI scores decreased by 5.15 points in the primary longitudinal analysis. The direction of this change persisted across the examined sensitivity scenarios, although statistical significance depended on assumptions about missing data.

**What are the implications of the main findings?**

- This study extends the limited evidence base for compounded 0.025% atropine by providing prospectively collected 24-month tear-film, symptom, and corneal endothelial outcomes in a European paediatric cohort.
- The findings are clinically reassuring for the measured outcomes in children completing follow-up with this formulation, while the completer-cohort design and absence of an untreated comparator preclude causal or population-wide tolerability conclusions.

**Abstract:**

**Background/Objectives:** Long-term tear-film and symptom data for compounded 0.025% atropine remain limited in European children. We assessed changes in first non-invasive tear-film break-up time (fNIBUT) and Ocular Surface Disease Index (OSDI) scores over 24 months. **Methods:** This secondary analysis of the ASPECT trial included 101 children completing 24 months, all assigned nightly compounded 0.025% atropine with Defocus Incorporated Multiple Segments or single-vision spectacles. Available fNIBUT, OSDI, and corneal endothelial cell density (ECD) measurements at baseline, 12 months, and 24 months were analysed using longitudinal mixed-effects models. The six pairwise contrasts across the primary outcomes of this secondary analysis used Holm-adjusted *p*-values and simultaneous Bonferroni 95% confidence intervals (CIs). No untreated comparator was available. **Results:** fNIBUT was numerically higher at 24 months than at baseline (16.9 vs. 14.3 s; mean difference [MD], 2.60 s; 95% CI, −0.37 to 5.57; *p* = 0.103). OSDI decreased from 14.7 to 9.6 (MD, −5.15 points; 95% CI, −9.93 to −0.36; *p* = 0.028). This reduction was not statistically significant in all examined missing-not-at-random scenarios or in an analysis restricted to children aged ≥8 years. ECD showed no statistically significant longitudinal changes. The exploratory combined criterion of OSDI ≥ 13 and fNIBUT < 10 s decreased numerically from 13/98 (13.3%) to 5/86 (5.8%). **Conclusions:** No deterioration was detected in the measured parameters among 24-month completers. Selection of completers, differential missingness, limited paediatric symptom assessment, and absence of a concurrent untreated comparator preclude firm conclusions about long-term ocular surface tolerability.

## 1. Introduction

Myopia is a major and growing public health concern because of its increasing prevalence and its association with sight-threatening complications, including myopic maculopathy, retinal detachment, glaucoma, and cataract [1,2]. Accordingly, interventions that limit excessive axial elongation during childhood may reduce the risk of long-term visual morbidity [3]. Low-concentration atropine is increasingly used for myopia control because it can slow refractive progression and axial elongation while causing fewer dose-related adverse effects, including photophobia, pupillary dilation, and near-vision difficulties, than higher concentrations [4,5,6].

Despite its increasing use as a long-term treatment in children, the effects of low-concentration atropine on the ocular surface remain incompletely characterised. Nightly administration over several years makes the assessment of tear-film stability, ocular symptoms, and treatment tolerability clinically relevant, as these factors may affect adherence and treatment persistence. Previous ocular surface studies have focused predominantly on atropine concentrations of 0.01% or 0.02% and have generally included relatively small samples with follow-up periods of 12 months or less [7,8,9]. The CHAMP trial evaluated 0.01% and 0.02% atropine in US and European children, and the Irish MOSAIC trial investigated 0.01% atropine in a predominantly White European population [10,11]. These trials provide relevant evidence on efficacy and general safety but do not resolve the specific question of 24-month tear-film stability and symptom trajectories during compounded 0.025% atropine use. Evidence specifically addressing compounded 0.025% atropine over longer treatment periods is therefore limited. This concentration warrants separate evaluation because ocular surface findings cannot necessarily be extrapolated across concentrations or formulations, particularly when compounded preparations may differ in their excipients and preservatives.

Assessing ocular surface changes in children receiving atropine is methodologically challenging [12]. Ocular surface symptoms and signs in children may also be associated with behavioural and environmental exposures, including digital device use and prolonged homework or near-work activities [13,14]. Longitudinal evaluation using both objective and patient-reported measures may therefore help identify clinically relevant changes during treatment. Nevertheless, in the absence of an untreated control group, such observations can characterise ocular surface trajectories and detect potential safety signals but cannot establish whether any changes—or absence of deterioration—are causally attributable to atropine.

The ASPECT randomised clinical trial compared Defocus Incorporated Multiple Segments (DIMS) and single-vision (SV) spectacle lenses in children receiving nightly compounded 0.025% atropine [15]. Although spectacle lenses do not directly contact the ocular surface, the optical intervention could indirectly influence ocular surface outcomes through changes in visual behaviour, near-work patterns, digital screen exposure, or spectacle-wearing adaptation. The randomised DIMS–SV comparison was therefore retained as an exploratory effect-modification analysis. Any between-group differences in ocular surface outcomes were therefore interpreted as exploratory and with appropriate caution regarding multiple comparisons.

The primary objective of this secondary analysis was to characterise 24-month changes in first non-invasive tear-film break-up time (fNIBUT) and Ocular Surface Disease Index (OSDI) scores in children with myopia receiving nightly compounded 0.025% atropine eye drops. The secondary objective was to assess longitudinal changes in corneal endothelial cell density (ECD). Exploratory objectives included evaluating the exploratory combined symptom and tear-film criterion; examining associations with SFChT, age, sex, and digital screen exposure; and comparing fNIBUT and OSDI outcomes between the randomised DIMS and single-vision spectacle groups.

## 2. Materials and Methods

### 2.1. Study Design and Participants

This study was a secondary longitudinal analysis of prospectively collected data from the ASPECT trial, conducted at Hospital Clínico San Carlos, Madrid, Spain. The parent trial randomised 111 participants. The source cohort for this analysis comprised the 101 participants who completed 24 months; the ten participants who withdrew earlier were excluded at all visits. Within this completer cohort, all available outcome-specific measurements at baseline, 12 months, and 24 months were analysed; valid measurements at all three visits were not required. The estimates therefore describe outcomes among completers rather than all randomised participants. Reporting was guided by applicable STROBE recommendations for cohort studies [16]; the randomised design and participant flow of the parent trial are described to clarify cohort selection [15]. The study adhered to the Declaration of Helsinki and was approved by the Ethics Committee of Hospital Clínico San Carlos (21/522-EC_M). The EudraCT record (2021-003373-64) was first entered on 27 July 2021. The study start date recorded in ClinicalTrials.gov is 23 February 2022. The additional ClinicalTrials.gov record (NCT06431841) was first submitted on 13 March 2024 and first posted on 29 May 2024, after recruitment had begun. Written informed consent was obtained from parents or legal guardians, with written assent from participants aged ≥12 years and verbal assent from younger children. Baseline ocular surface measurements preceded the first atropine administration.

### 2.2. Inclusion and Exclusion Criteria

Eligible participants were children aged 4–16 years with myopia, defined as a cycloplegic spherical equivalent refraction (SER) between −1.00 and −6.00 dioptres (D) in each eye, astigmatism ≤ 2.00 D, and an absolute interocular difference in SER of ≤1.50 D. SER was calculated as the spherical power plus one-half of the cylindrical power. Additional eligibility criteria included a best-corrected visual acuity of ≤0.02 logMAR in each eye and the ability and willingness of participants and their parents or legal guardians to comply with the study procedures. Exclusion criteria comprised strabismus or binocular vision disorders, previous ocular surgery, ocular or systemic conditions potentially affecting visual function or ocular growth, neurological or developmental disorders precluding reliable examination, and previous use of any pharmacological or optical myopia-control intervention.

### 2.3. Interventions

Participants were randomised in a 1:1 ratio to receive either single-vision or Defocus Incorporated Multiple Segments (DIMS) spectacle lenses. Details of the randomisation and allocation procedures have been reported previously [15]. All participants were instructed to instil one drop of compounded 0.025% atropine into each eye once nightly throughout the 24-month study period.

All atropine preparations were compounded and dispensed by the same licensed pharmacy. According to information provided by the pharmacy, the nominal atropine concentration was 0.025%, with an aqueous saline vehicle, acetate buffer, cetrimide preservative, and a reported pH of 5.0–6.0. The exact cetrimide concentration, osmolarity, and batch-specific analytical quality-control results for atropine content, pH, and osmolarity were not available to the investigators. This does not establish that such quality-control testing was not performed. The stated beyond-use periods were 90 days unopened and 30 days after opening, with storage at room temperature or under refrigeration according to pharmacy instructions. Adherence was assessed indirectly using dispensing records, caregiver questionnaires, and telephone follow-up; objective dose-by-dose adherence and daily spectacle wear time were unavailable.

### 2.4. Clinical Assessment

Participants attended parent-trial visits at baseline and 6, 12, 18, and 24 months. Ocular surface and corneal assessments were scheduled at baseline, before treatment, and at 12 and 24 months. Follow-up ocular surface examinations were conducted between 10:00 a.m. and 1:00 p.m., following atropine administration on the preceding evening before 11:00 p.m. This schedule implies an interval of at least approximately 11 h. Individual instillation times were not recorded, so the exact interval could not be determined for each participant.

At each study visit, monocular and binocular visual acuity were assessed using standardised Early Treatment Diabetic Retinopathy Study (ETDRS) charts and recorded in logarithm of the minimum angle of resolution (logMAR) units. Subjective refraction was performed at distance and near according to standard clinical procedures. Binocular vision assessment included cover testing at distance and near, measurement of the near point of convergence, assessment of stereopsis using the TNO stereotest, and evaluation of sensory fusion using the Worth Four Dot test. Participants also completed age-appropriate quality-of-life questionnaires as part of the study protocol.

Tear-film stability was assessed using the Oculus Keratograph 5M (Oculus Optikgeräte GmbH, Wetzlar, Germany). The analysed metric was NIKBUT-first, termed first non-invasive tear-film break-up time (fNIBUT), representing the interval from the last blink to the first detected tear-film break-up. Because repeated measurements were challenging in children, one technically acceptable recording was retained per assessment. Technically inadequate acquisitions were repeated where feasible; multiple valid recordings were not averaged, and NIKBUT-average was not analysed. When cooperation prevented an acceptable recording, fNIBUT was unavailable. Ocular symptoms were assessed with the OSDI, scored from 0 to 100. Parents or guardians could assist younger children in understanding the items, but assistance was not systematically recorded and could not be quantified retrospectively. Unfilled questionnaires were excluded. ECD was assessed using non-contact specular microscopy (EM-4000, Tomey Corporation, Nagoya, Japan). Central corneal thickness was measured with Scheimpflug tomography (Pentacam, OCULUS Optikgeräte GmbH, Wetzlar, Germany) as part of the parent protocol but was not analysed here.

Cycloplegic autorefraction was performed using an autorefractor/keratometer (HRK-7000A, Huvitz Co., Ltd., Gunpo, Republic of Korea). Axial length (AL) was measured using optical low-coherence interferometry (Lenstar LS 900, Haag-Streit AG, Köniz, Switzerland). Spectral-domain optical coherence tomography with enhanced depth imaging (Spectralis OCT, Heidelberg Engineering, Heidelberg, Germany) was used to measure subfoveal choroidal thickness (SFChT). Anterior segment biomicroscopy, dilated fundus examination, and intraocular pressure measurement using rebound tonometry (iCare IC100, Icare Finland Oy, Helsinki, Finland) were performed at all study visits.

Screen-exposure assessment was introduced after recruitment had begun; the earliest enrolled participants were therefore not administered the questionnaire. All screen-exposure responses used here were collected at baseline. Information included school screen use and average daily weekday and weekend exposure, categorised as <2 h, 2–4 h, or >4 h. Descriptive and exploratory analyses used available responses for each variable; missing responses were not classified as no screen exposure. Ocular and systemic adverse events were elicited during follow-up, including their relationship to treatment and actions taken. The safety summary available for this secondary analysis did not provide separate counts for every symptom.

All examinations were conducted by trained examiners under routine clinical conditions using standardised procedures. For eye-specific analyses, only one eye per participant was included to avoid inter-eye correlation. The right eye was selected by default; when image quality or examination reliability was insufficient in the right eye, the fellow eye was selected. The same selected eye was retained across all follow-up visits. Further details of the complete ASPECT examination protocol have been reported previously [15].

### 2.5. Outcomes

fNIBUT, OSDI, and ECD were collected prospectively as protocol-defined assessments. Their designation as the primary outcomes (fNIBUT and OSDI) and a secondary outcome (ECD) of the present secondary analysis was established retrospectively and was not prespecified in the parent trial protocol or registry. The public EudraCT record does not separately list these three measurements as secondary endpoints. The combined criterion of OSDI ≥ 13 and fNIBUT < 10 s was exploratory and used adult-derived thresholds, rather than constituting a validated paediatric diagnosis. Additional exploratory analyses examined associations with SFChT, baseline age, sex, and baseline screen exposure, and visit-specific differences between DIMS and SV groups.

Treatment discontinuations and reported adverse events were recorded throughout follow-up, with particular attention to ocular surface-related events; these data provide descriptive information rather than a comprehensive assessment of safety or tolerability.

### 2.6. Statistical Analysis

Categorical variables are reported as *n* (%) and quantitative variables as mean ± standard deviation (SD), or median [interquartile range] where appropriate. Longitudinal continuous outcomes were analysed using linear mixed-effects models with categorical visit and a participant-specific random intercept, incorporating all available outcome-specific observations within the 101-participant completer cohort. Likelihood-based inference assumes missingness is at random conditional on the model; this assumption cannot be verified. The combined symptom and tear-film criterion was evaluated only when both components were available and analysed using generalised estimating equations (GEE), with a binomial family, logit link, exchangeable working correlation, and robust standard errors. GEE estimates are population-averaged probabilities and between-visit odds ratios (ORs). The small number of events limits precision.

Baseline-age-by-visit interactions were fitted to assess age-related differences in trajectories. The OSDI age model was independently recalculated using restricted maximum likelihood, with categorical visit, continuous baseline age, their interaction, and a participant-specific random intercept. Individual coefficients used two-sided normal Wald tests and unadjusted 95% CIs; the global age-by-visit interaction used a two-degree-of-freedom Wald chi-square test. Spectacle-group and sex contrasts were estimated at each visit. The exploratory fNIBUT–SFChT association was assessed using visit-specific Pearson correlations among participants with both measurements available. Table A1 (Appendix A) reports correlation coefficients, confidence intervals, and *p*-values from the revised statistical output; the 12-month *p*-value was recalculated from the reported rounded r and *n* using the conventional two-sided Pearson *t* test (see table note). Regression slopes are not presented. Baseline associations with age were described using Spearman correlations. Statistical significance was assessed at 0.05. Original analyses used R 4.5.1 and IBM SPSS Statistics 26.0; the OSDI age-model recalculation and supplementary imputation analysis used Python 3.12 with NumPy 2.3.5 and SciPy 1.17.0.

No a priori sample-size calculation was performed for these secondary outcomes; the sample was determined by the parent trial and availability of follow-up. The six pairwise visit contrasts across the two primary outcomes of this secondary analysis formed one multiplicity family: *p*-values were adjusted using Holm’s method and confidence intervals using Bonferroni simultaneous 95% coverage. Per-visit mean CIs were unadjusted. For ECD and the combined criterion, adjustment was across the three visit contrasts; for spectacle comparisons, across three visits within each outcome. For sex comparisons, the statistical report specifies Holm-adjusted *p*-values and Bonferroni 95% CIs across the three visit-specific comparisons within each outcome (Table A2). For weekday and weekend screen exposure, Holm *p*-value and Bonferroni CI adjustment applied to three category contrasts within each visit and outcome; school use had one contrast per visit. Global interaction tests and individual age coefficients were unadjusted. These within-family adjustments do not control multiplicity across all exploratory analyses.

To examine OSDI reporting in younger children, the longitudinal analysis was repeated among the 85 participants aged ≥8 years at baseline (Table A3). An analysis restricted to unassisted questionnaires was not possible because assistance was not recorded.

A supplementary overall-cohort OSDI sensitivity analysis used 2000 predictive mean matching imputations, with five donors, 20 iterations, and seed 20260928. Predictors were baseline OSDI, the other follow-up OSDI, baseline age, and sex. The sensitivity analysis pooled the cohort and did not include spectacle allocation; it therefore does not assess robustness of the DIMS–SV comparison. After imputation under a missing-at-random (MAR) reference, positive delta shifts were added to originally missing scores at 12 and 24 months and capped at 100. Each completed dataset was analysed using a visit-plus-random-intercept REML model. Results were pooled using Rubin’s rules with Barnard–Rubin degrees of freedom (complete-data degrees of freedom set to 100). A conservative Bonferroni correction for six contrasts was applied to *p*-values and CIs within each scenario, distinct from the primary Holm procedure. Delta values of 0, 2.5, 5, and 10 points illustrate sensitivity to departures from MAR [17]. A supplementary scenario shifted only 24-month missing values (Table A4).

An additional descriptive analysis used paired baseline and 24-month right-eye photopic and mesopic pupil diameters, without imputation, to contextualise possible residual pharmacological effects. Values and within-participant changes are presented as mean ± SD.

## 3. Results

### 3.1. Participant Flow and Baseline Characteristics

**Figure 1:**
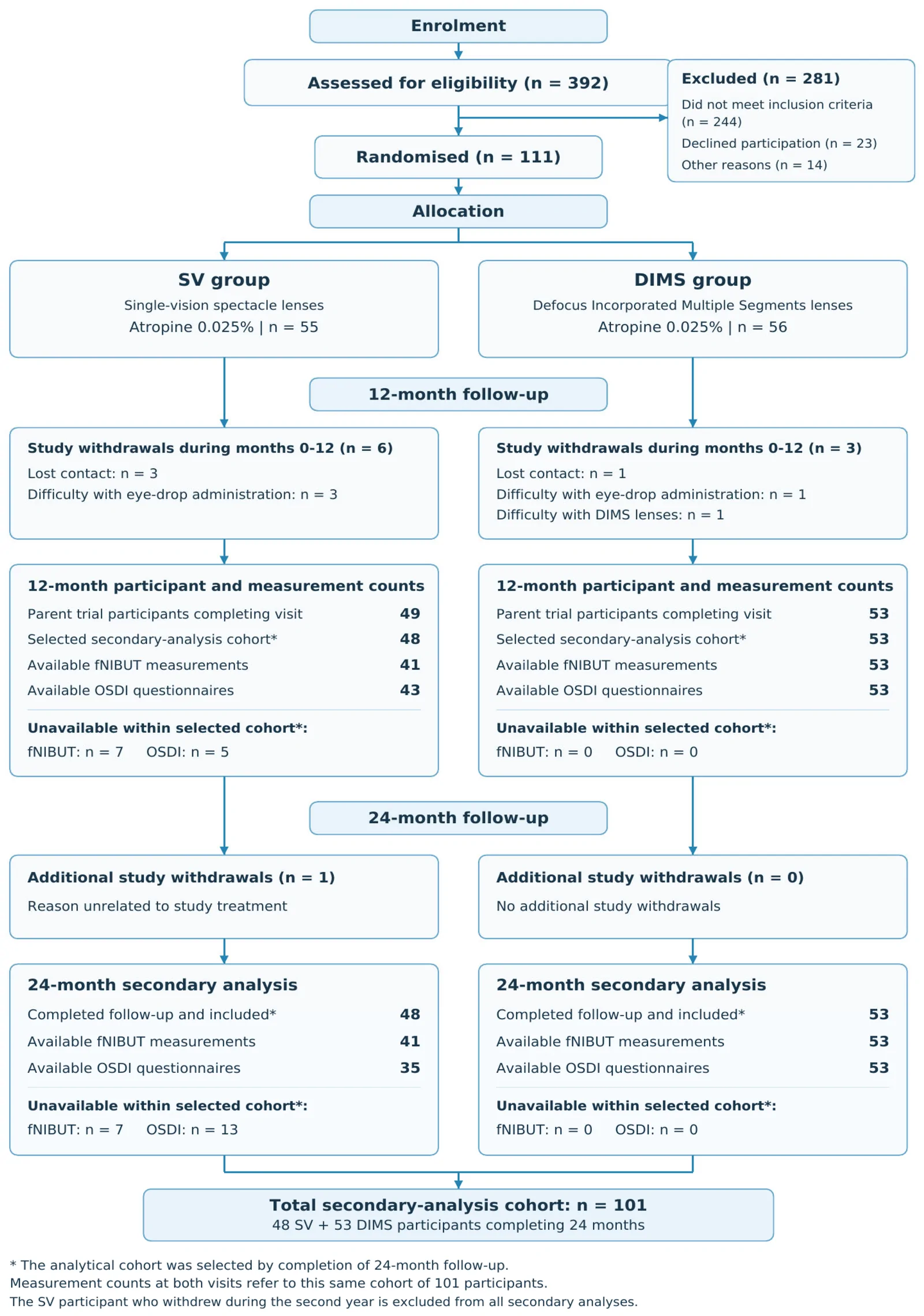
Participant flow in the ASPECT trial and selection of the secondary-analysis cohort. Counts of available and unavailable outcomes refer to the same 101 completers at both follow-up visits. The SV participant who withdrew in the second year is excluded from all secondary analyses. SV, single-vision; DIMS, Defocus Incorporated Multiple Segments; fNIBUT, first non-invasive tear-film break-up time; OSDI, Ocular Surface Disease Index.

Of 111 randomised participants, 55 received SV and 56 DIMS spectacles (Figure 1). First-year withdrawals comprised loss of contact (three SV, one DIMS), difficulty administering or accepting the drops (three SV, one DIMS), and difficulty using DIMS spectacles (one DIMS). Thus, 49 SV and 53 DIMS participants completed the parent trial’s 12-month visit. One additional SV participant withdrew during the second year for a reason unrelated to treatment. The present cohort therefore comprised 48 SV and 53 DIMS participants completing 24 months. Administration-related withdrawals involved practical difficulties or refusal of instillation, with minimal exposure in several cases. One SV child complained about instillation and refused further doses; the parent stopped treatment after approximately two days. This child is included among the three SV drop-administration withdrawals, not an additional withdrawal. No specific ocular surface manifestation was documented as the reason. Refusal may nonetheless reflect discomfort or poor acceptability.

Measurements were recoverable for five of the ten excluded participants: three had baseline and 12-month measurements, and two had baseline measurements only; none had 24-month measurements. Apart from the participant who later withdrew during the second year, the other nine were no longer receiving atropine at 12 months. Available measurements after treatment cessation were not included in the completer analysis. Two DIMS participants requiring photochromic lenses for photophobia remained in follow-up.

Within this 101-participant completer cohort, the availability of valid ocular surface measurements varied according to outcome and study visit. Valid fNIBUT measurements were available for 98 participants at baseline, 94 at 12 months, and 94 at 24 months, whereas OSDI scores were available for 101, 96, and 88 participants, respectively. Paired baseline and 24-month measurements were available for 91 participants for fNIBUT and 88 for OSDI. The exploratory combined symptom and tear-film instability criteria could be evaluated in 98 participants at baseline, 93 at 12 months, and 86 at 24 months. All available outcome-specific observations within the completer cohort were included in the corresponding mixed-effects models; complete ocular surface data at all three assessment visits were not required. Figure 1 presents both participant attrition in the parent ASPECT trial and outcome-specific data availability for the present secondary analysis. Baseline demographic and ocular characteristics of the source cohort are summarized in Table 1. Missing follow-up fNIBUT measurements reflected insufficient cooperation to obtain an acceptable recording, and missing OSDI values reflected unfilled questionnaires. Within this cohort, unavailable follow-up measurements occurred in SV: seven fNIBUT measurements at each follow-up, and five and thirteen OSDI questionnaires at 12 and 24 months. The reason for this concentration in one group could not be established. Baseline SER and AL were available in 96 participants (−2.26 ± 1.19 D and 24.40 ± 0.88 mm). Screen data were available for 73 participants for school use and weekdays and 72 for weekends.

### 3.2. Longitudinal Changes in fNIBUT and OSDI

**Figure 2:**
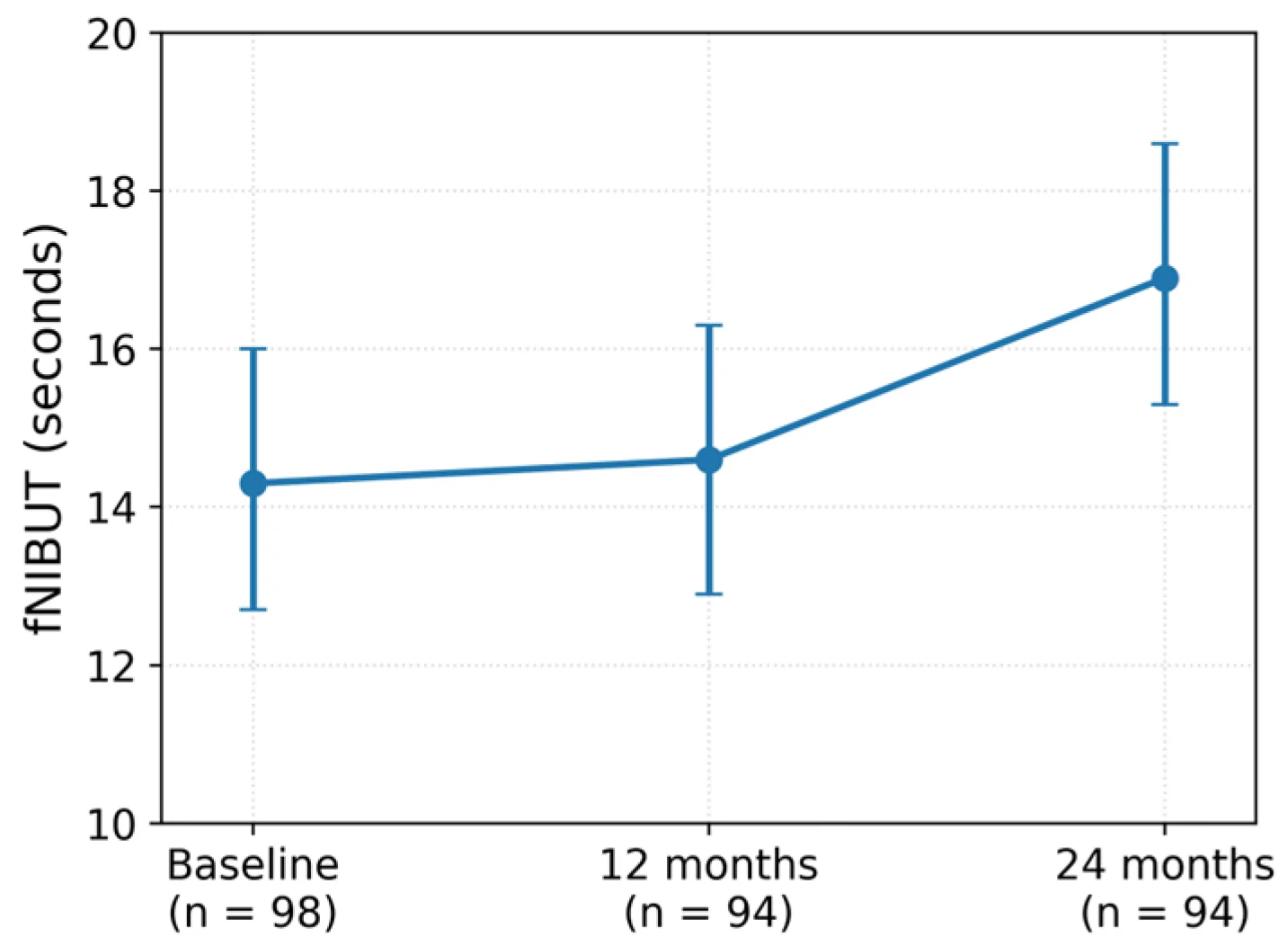
Model-estimated fNIBUT means with unadjusted 95% CIs. The number of available observations is shown beneath each visit. No pairwise contrast was statistically significant after multiplicity adjustment (Table 2).

**Figure 3:**
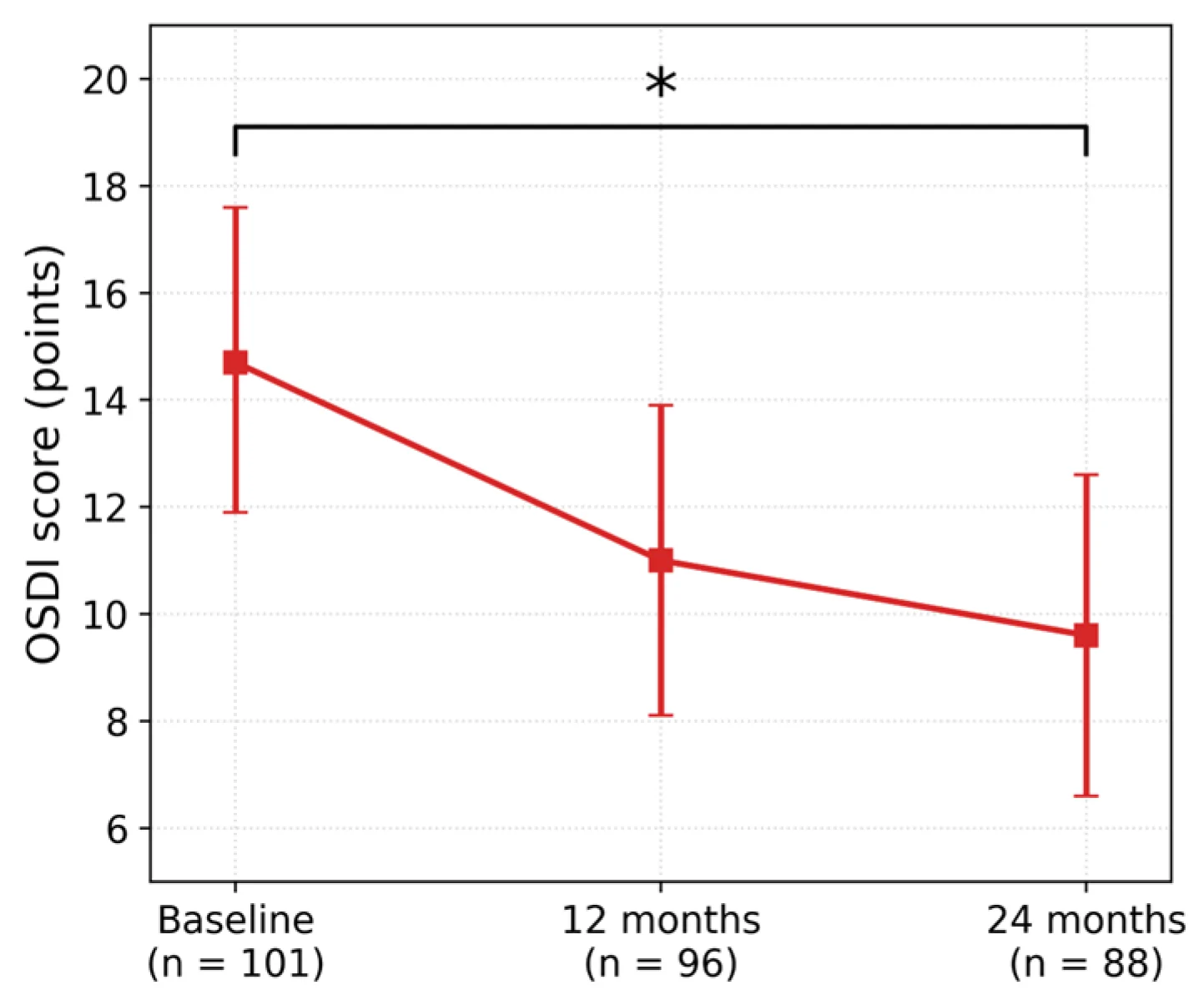
Model-estimated OSDI means with unadjusted 95% CIs. Higher scores indicate greater symptom severity. Available observations are shown beneath each visit. * Baseline versus 24 months: Holm-adjusted *p* = 0.028; other contrasts were not statistically significant (Table 2).

fNIBUT showed no statistically significant longitudinal changes (Table 2; Figure 2). Differences were 0.25 s at 12 months versus baseline (95% CI, −2.72 to 3.21; *p* = 0.868), 2.60 s at 24 months versus baseline (95% CI, −0.37 to 5.57; *p* = 0.103), and 2.35 s at 24 versus 12 months (95% CI, −0.64 to 5.35; *p* = 0.138). OSDI was lower at 24 months than baseline (MD, −5.15 points; 95% CI, −9.93 to −0.36; *p* = 0.028), whereas the baseline-to-12-month (MD, −3.72; 95% CI, −8.38 to 0.94; *p* = 0.138) and 12-to-24-month differences (MD, −1.43; 95% CI, −6.27 to 3.42; *p* = 0.868) were not statistically significant (Figure 3).

In the overall OSDI imputation analysis under MAR, the 24-month difference was −5.04 points (Bonferroni 95% CI, −9.84 to −0.24; *p* = 0.034). Adding 2.5 points to each originally missing follow-up score yielded a difference of −4.72 (95% CI, −9.53 to 0.09; *p* = 0.057). Larger shifts further weakened statistical evidence. Shifting only missing 24-month values produced similar findings (Table A4). Thus, the apparent reduction was not statistically significant under all examined missing-not-at-random assumptions.

### 3.3. Corneal Endothelial Cell Density

No statistically significant longitudinal change in endothelial cell density was detected. Mean values were 3015.32 ± 316.95 cells/mm^2^ at baseline, 3055.00 ± 237.56 cells/mm^2^ at 12 months, and 3045.42 ± 242.02 cells/mm^2^ at 24 months. No statistically significant differences were observed at 12 months (95% CI, −18.5 to 94.3; *p* = 0.319) or 24 months (95% CI, −30.3 to 81.0; *p* = 0.544) compared with baseline, or between the 12- and 24-month visits (95% CI, −67.3 to 42.2; *p* = 0.581) (Table A5). Baseline ECD could not be obtained in 11 children because of measurement difficulties. The denominators of 90, 95, and 98 reflect visit-specific availability within the same cohort.

### 3.4. Exploratory Analyses

#### 3.4.1. Combined Symptom and Tear-Film Criterion

The exploratory combined criterion was met by 13/98 participants (13.3%) at baseline, 8/93 (8.6%) at 12 months, and 5/86 (5.8%) at 24 months. GEE population-averaged probabilities were 13.2% (95% CI, 7.8–21.5%), 8.8% (4.5–16.5%), and 5.9% (2.5–13.3%), respectively. No adjusted pairwise comparison was statistically significant (Table A6).

#### 3.4.2. Spectacle Group Comparison

Model-estimated fNIBUT and OSDI scores did not differ significantly between the DIMS (*n* = 53) and SV (*n* = 48) groups at baseline, 12 months, or 24 months (all *p* > 0.05; Table A7).

#### 3.4.3. Subfoveal Choroidal Thickness

Pearson correlations between SFChT and fNIBUT were weak at baseline (r = 0.044; *n* = 91), 12 months (r = 0.180; *n* = 90), and 24 months (r = 0.013; *n* = 83). The corresponding *p*-values were 0.680, approximately 0.09, and 0.909, respectively; none was statistically significant (Table A1).

#### 3.4.4. Age and Sex

**Figure A1:**
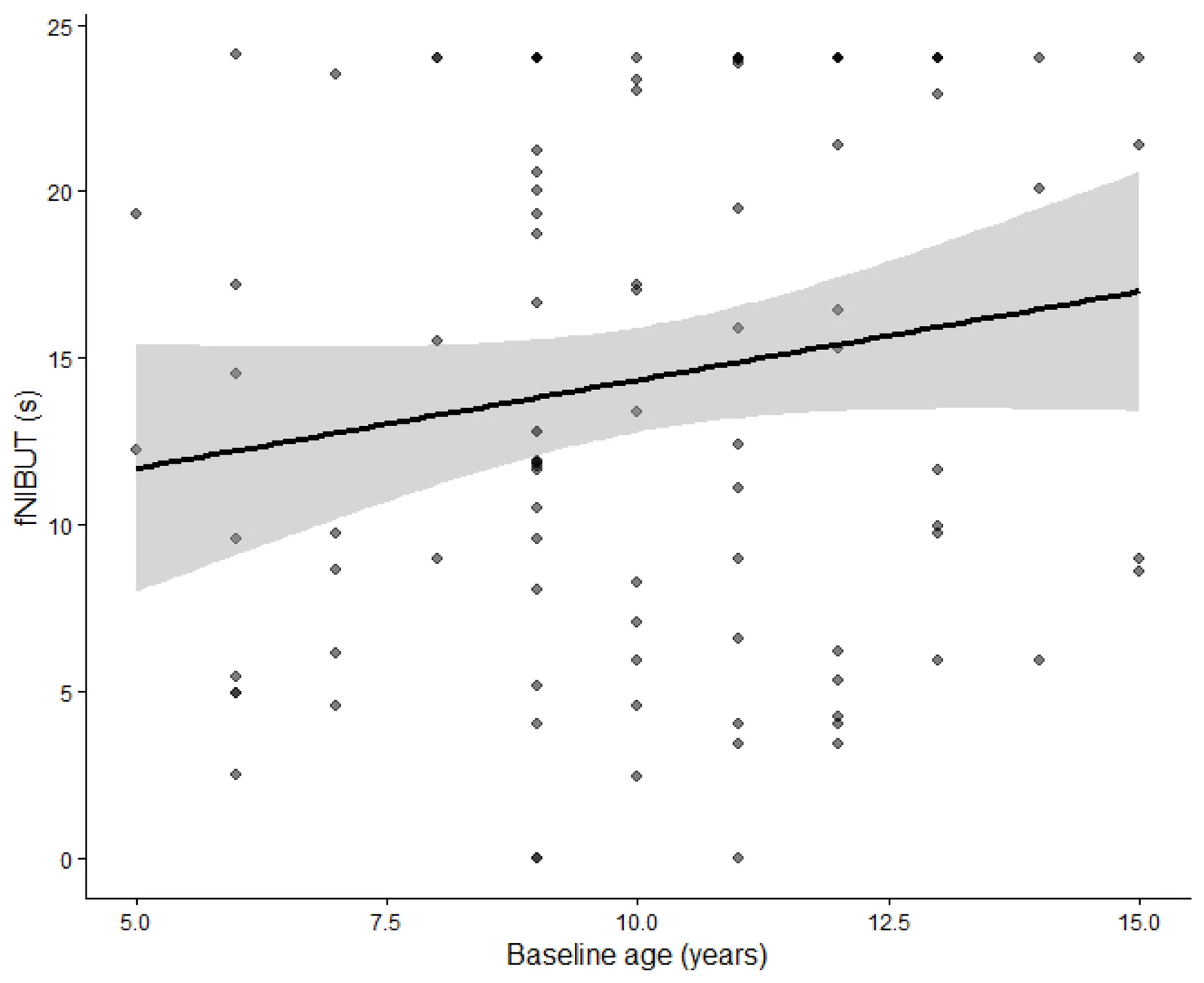
Baseline age and fNIBUT. Spearman ρ = 0.14; *p* = 0.16. Points represent available baseline observations. The line and shaded band are visual regression summaries; the reported association statistic is Spearman’s rank correlation. A non-significant result does not establish absence of an age effect.

**Figure A2:**
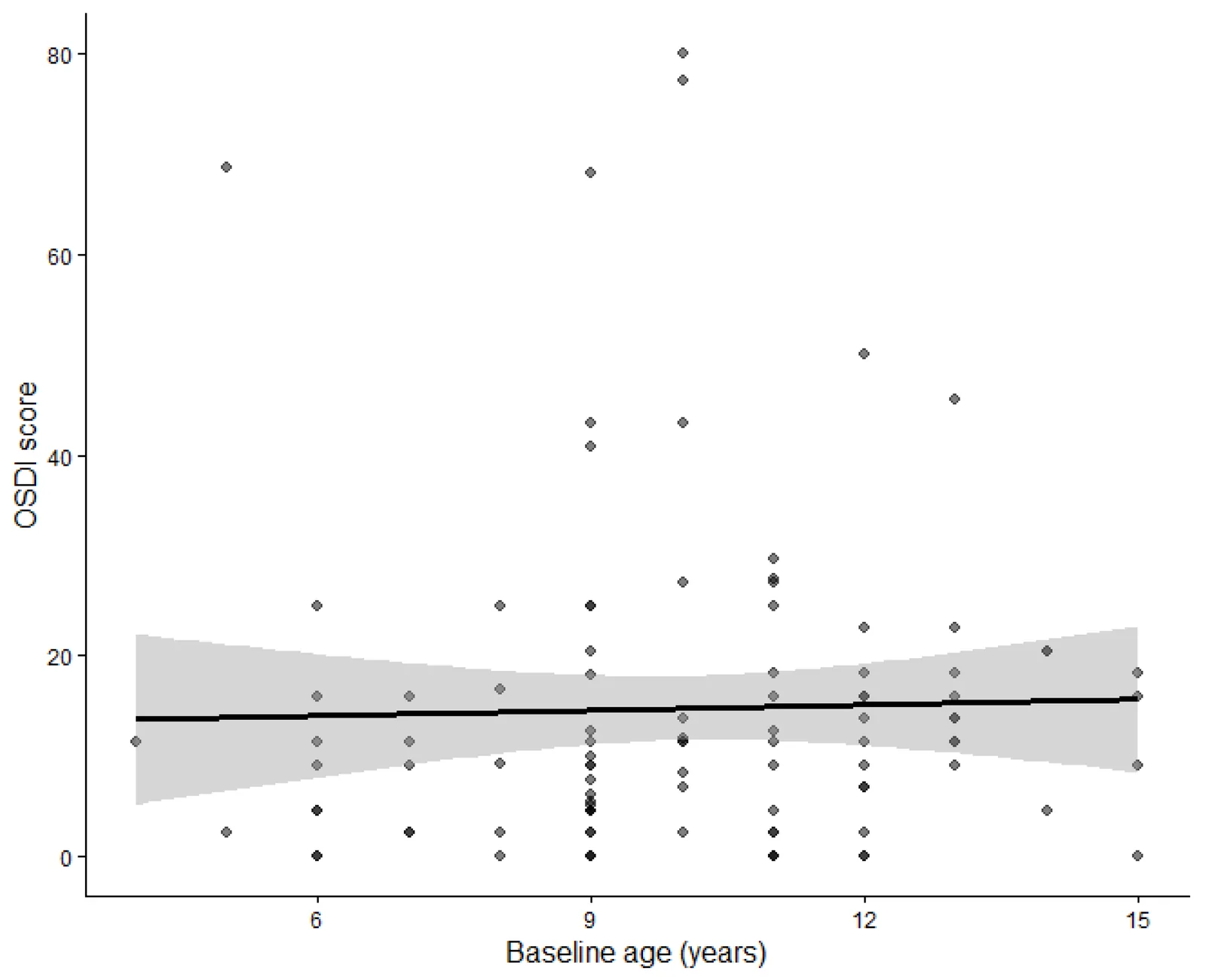
Baseline age and OSDI. Spearman ρ approximately 0.17; *p* = 0.09. Points represent available baseline observations. The line and shaded band are visual regression summaries; the reported association statistic is Spearman’s rank correlation. A non-significant result does not establish absence of an age effect.

No statistically significant baseline-age associations or age-by-visit interactions were detected (Table A8). Baseline-age coefficients were 0.56 s/year for fNIBUT (95% CI, −0.13 to 1.25; *p* = 0.112) and 0.18 OSDI points/year (95% CI, −0.98 to 1.35; *p* = 0.758). Global interaction *p*-values were 0.640 and 0.458, respectively. Sixteen children (15.8%) were younger than 8 years. Among the 85 aged ≥8 years, the 24-month OSDI reduction was −5.40 points (95% CI, −10.98 to 0.19; adjusted *p* = 0.064; Table A3). The direction was similar to the primary result, but statistical significance was not retained. Baseline Spearman correlations with age were weak (fNIBUT: ρ = 0.14, *p* = 0.16; OSDI: ρ approximately 0.17, *p* = 0.09; Figure A1 and Figure A2).

No statistically significant differences according to sex were observed for either fNIBUT or OSDI at baseline, 12 months, or 24 months. Model-estimated differences between girls and boys were small at all visits, with all 95% CIs including zero (all *p* > 0.05) (Table A2).

#### 3.4.5. Screen Exposure

Exploratory analyses showed no consistent association between reported screen exposure and either fNIBUT or OSDI scores. No statistically significant differences were observed according to school-based screen use or weekend screen time. At baseline, participants reporting 2–4 h of weekday screen exposure had higher OSDI scores than those reporting < 2 h (mean difference, 9.41 points; 95% CI, 0.39–18.44; *p* = 0.038). However, this isolated difference was not observed at 12 or 24 months and was not accompanied by a dose–response pattern (Table A9).

### 3.5. Reported Adverse Events and Treatment Discontinuations

Clinical examinations included BCVA, accommodative amplitude, photopic and mesopic pupil diameter, slit-lamp biomicroscopy, posterior-segment examination, and adverse-event recording. The available 24-month safety summary is presented in Table A10. It includes one early SV atropine discontinuation associated with refusal of instillation and two DIMS participants prescribed photochromic lenses at 6 months for photophobia. The latter remained in follow-up. No additional treatment-related discontinuations occurred during the second year, and no serious ocular or systemic adverse events were reported. Separate counts for every symptom were unavailable; absence of an event from the table is not evidence of zero incidence.

Paired right-eye photopic pupil measurements were available for 81 participants: 3.68 ± 0.70 mm at baseline and 5.13 ± 0.92 mm at 24 months, with a within-participant change of +1.45 ± 0.96 mm. Paired mesopic measurements were available for 80 participants: 6.60 ± 0.89 mm and 7.30 ± 0.61 mm, respectively, with a change of +0.71 ± 0.83 mm. These descriptive summaries apply to participants with paired measurements.

## 4. Discussion

This secondary analysis describes tear-film stability, ocular symptoms, and ECD among 101 children completing 24 months of the ASPECT trial. No statistically significant fNIBUT or ECD changes were detected. The primary OSDI reduction was sensitive to missing-data assumptions and did not retain statistical significance in children aged ≥8 years. The combined criterion decreased numerically, with few events and substantial uncertainty. No significant DIMS–SV differences were detected, but differential missingness limits that comparison. These findings do not establish long-term tolerability for all children starting atropine.

Our findings on tear-film stability are consistent with and extend those of previous shorter-term studies evaluating low-concentration atropine. Cheng et al. observed no significant changes in either the first or average non-invasive keratographic break-up time (reported as NK-BUTfirst and NK-BUTave) after six months of treatment with 0.01% atropine [7]. Li et al. likewise found no significant longitudinal changes in fNIBUT or average non-invasive break-up time over 12 months of treatment with 0.01% or 0.02% atropine [8]. Zhao et al., using conventional tear-film break-up time (TBUT), found no significant differences attributable to 0.01% atropine after 12 months [9]. Cho et al. measured both first and average non-invasive break-up times using the Keratograph 5M and found no differences among treatment-naïve children and those managed with atropine, orthokeratology, or their combination [18]. Although the assessment methods were not identical, these studies did not demonstrate a consistent deterioration in tear-film stability during low-concentration atropine treatment. By extending follow-up to 24 months and specifically evaluating fNIBUT, our study provides longer-term evidence in European children treated with a compounded 0.025% formulation.

Considerable heterogeneity exists in the literature regarding tear-film stability assessment, as studies have used either conventional fluorescein-based tear-film break-up time (TBUT) or non-invasive measurements such as fNIBUT. Although both are established clinical approaches, their values are not directly interchangeable [19,20]. In accordance with the TFOS DEWS II diagnostic recommendations, fNIBUT was selected because of its objective and non-invasive nature, which is particularly advantageous in paediatric populations [21]. In our cohort, fNIBUT showed no statistically significant longitudinal change, although a modest numerical increase was observed at 24 months. Because assessments were scheduled at 12-month intervals, each participant was generally examined during a similar period of the year, which may have reduced seasonal variability; however, an influence of season cannot be completely excluded. Environmental conditions such as temperature and humidity were not systematically recorded or incorporated into the statistical models; therefore, residual environmental effects cannot be ruled out. The numerical increase in fNIBUT could reflect developmental changes, greater familiarity with the examination procedure, measurement variability, or a true biological change. As the present study was not designed to distinguish among these explanations, this finding should be interpreted as an absence of evidence of deterioration in tear-film stability rather than evidence of treatment-related improvement.

Monitoring ocular symptoms in children receiving long-term atropine treatment is important for detecting potential adverse effects, maintaining patient comfort, and supporting treatment adherence. However, validated dry eye questionnaires specifically designed for paediatric populations remain scarce, and children may experience greater difficulty than adults in identifying, interpreting, and reporting ocular symptoms [22,23,24]. Although the OSDI was originally developed for adult populations and is among the symptom questionnaires recommended by TFOS DEWS II [21], it has been widely used in paediatric ocular surface research [12,25,26,27,28,29] with evidence on repeatability in some paediatric age groups [12]. This does not establish validity in very young children, including 4-year-olds. Nevertheless, developmental differences in comprehension and unrecorded parental assistance should be considered when interpreting paediatric OSDI scores. These limitations reinforce the need to develop and validate age-appropriate instruments for assessing ocular surface symptoms in children [22,30,31].

Mean OSDI declined from 14.7 at baseline to 11.0 and 9.6 at follow-up. Similar studies at 0.01% or 0.02% have reported no increase in symptoms [7,8]. However, the primary 24-month reduction should be interpreted cautiously: a positive shift of only 2.5 points in missing follow-up values removed statistical significance in the sensitivity procedure. The ≥8-year analysis also had an interval including zero. Conventional OSDI cut-offs are adult-derived, and group means cannot represent individual clinical trajectories. Regression to the mean, developmental changes, familiarity with the questionnaire, parental assistance, and selective non-response could contribute. The findings do not establish symptom improvement attributable to atropine.

ECD showed no statistically significant longitudinal change. A meta-analysis comparing orthokeratology with and without 0.01% atropine similarly reported no significant ECD difference [32], although the lower concentration, concurrent intervention, and shorter follow-up limit comparability. In vitro atropine cytotoxicity has been reported [33], but those exposure conditions cannot be directly extrapolated to nightly topical treatment. Absence of a detected change in this selected cohort does not establish endothelial safety in all treated children.

As an exploratory analysis, we applied the adult diagnostic criteria recommended by the TFOS DEWS II, which combine both signs and symptoms [21]. In our study, participants were considered to meet the combined criteria when they concurrently had an OSDI score ≥ 13 and an fNIBUT < 10 s. The proportion meeting these criteria decreased numerically from 13.3% (13/98) at baseline to 8.6% (8/93) at 12 months and 5.8% (5/86) at 24 months, although the longitudinal difference was not statistically significant. Clear diagnostic thresholds for ocular surface tests in children have not yet been established, prompting the use of adult-derived criteria in this study and limiting their interpretation in a paediatric population. To our knowledge, few studies investigating low-dose atropine in children have evaluated a combined sign-and-symptom outcome rather than isolated ocular surface parameters. Independent of refractive status, the pooled prevalence of paediatric dry eye disease has been estimated at 23.7%, although estimates differ substantially between studies based on clinical signs (16.6%) and those based on symptom questionnaires (34.6%) [13,14]. This heterogeneity may reflect the recognised discordance between dry eye signs and symptoms [22,31], compounded in children by difficulties in symptom comprehension and reporting [12,22,30]. The few events and wide GEE intervals preclude concluding that atropine reduces dry eye occurrence. A validated paediatric multimodal diagnostic framework remains needed.

A growing body of evidence has linked DED with myopia in adults [34,35] and adolescents [36], with some studies suggesting a similar relationship in younger children [37,38,39]. However, much of this evidence is cross-sectional, limiting conclusions regarding the direction or causality of this relationship. The proportion of children with an OSDI score ≥13 in our study at baseline (40/101; 39.6%) was higher than the pooled questionnaire-based prevalence of 34.6% reported by Zou et al. [13]. Nevertheless, this comparison should be considered descriptive because the meta-analysis included heterogeneous populations, questionnaires, and diagnostic thresholds, whereas our cohort comprised exclusively myopic children evaluated using an adult-derived OSDI threshold.

In myopic children with dry eye symptoms, Hazra et al. found that a shorter fluorescein TBUT was associated with a longer AL and a thinner SFChT; SFChT was positively associated with TBUT and negatively associated with AL [37]. The authors proposed that shared parasympathetic regulation of the lacrimal glands and choroid might contribute to the relationship between DED and myopia. In contrast, we found no significant associations between fNIBUT and SFChT. Several clinical and methodological differences may explain these discrepant findings. The cohort studied by Hazra et al. consisted exclusively of symptomatic children and had a higher mean baseline myopia than our cohort (−4.61 ± 2.40 D vs. −2.26 ± 1.19 D). Furthermore, they assessed tear-film stability using fluorescein TBUT, whereas we used objective, non-invasive fNIBUT. Their cross-sectional design also precluded conclusions regarding causality. Further prospective studies are required to determine whether DED is a cause, consequence, or coexisting condition in childhood myopia, particularly among children receiving long-term topical treatments for myopia control.

Digital device use is widely recognised as a modifiable risk factor for DED in both adults and children [40,41,42], partly through reduced blink frequency and a greater proportion of incomplete blinks, which may compromise tear-film stability [43]. In our cohort, children reporting 2–4 h of weekday screen exposure at baseline had higher OSDI scores than those reporting < 2 h (mean difference, 9.41 points; 95% CI, 0.39–18.44; *p* = 0.038). However, this isolated difference was not observed at 12 or 24 months, was not accompanied by a dose–response pattern, and no consistent association was found between reported screen exposure and fNIBUT. These findings partly align with those of Kazanci and Eroglu, who reported a weak positive correlation between screen exposure and OSDI scores in healthy children but no correlation with tear break-up time or other objective ocular surface measures [44]. Given the exploratory nature of our analyses, the reliance on self-reported categorical exposure, and the multiple subgroup comparisons performed, the baseline association should be interpreted cautiously and may reflect residual confounding or chance. Further longitudinal studies using objective measures of screen exposure and blinking behaviour are needed to clarify the effects of digital device use on the paediatric ocular surface. Screen data were unavailable for the earliest recruits because the questionnaire was introduced later; this may have introduced selection related to recruitment period.

The documented adverse events and treatment discontinuations included an early atropine discontinuation associated with difficulty accepting instillation and two cases of photophobia requiring photochromic DIMS lenses. No serious ocular or systemic adverse events were reported in the 24-month safety summary. However, that summary does not provide a complete symptom-specific breakdown, and interventions cannot be equated with the incidence of all symptoms. Direct comparisons with adverse-event rates from other atropine trials are therefore not warranted.

Cetrimide has shown concentration-dependent cytotoxicity in conjunctival epithelial cell models [45]. The relevance to this cohort cannot be assessed without the preservative concentration and exposure characterisation. Compounded atropine preparations may vary in active content, pH, osmolarity, and excipients [46]. Although one pharmacy supplied the study, the exact cetrimide concentration, osmolarity, and batch-specific analytical results were unavailable. Thus, formulation consistency could not be analytically verified and the findings cannot establish formulation-specific safety. No vehicle or preservative-free control was available to separate atropine from excipient effects or support generalisation to other preparations.

### Strengths and Limitations

This study provides 24-month data on complementary tear-film, symptom, and corneal endothelial measurements in European children receiving compounded 0.025% atropine. Assessments were collected prospectively at protocol-defined visits. However, the hierarchy and analyses were defined subsequently and should not be interpreted as a prespecified confirmatory evaluation. The parent trial’s randomisation permits exploratory spectacle comparisons, but it does not supply an untreated atropine control.

Restricting the analysis to 24-month completers introduces selection and survivorship bias, particularly because administration or acceptance difficulties contributed to withdrawals. Excluding observations after treatment cessation does not remove this bias. Mixed models can use incomplete observations, but the present analysis was deliberately restricted to completers and was not rerun in all 111 randomised participants. The absence of an additional analysis incorporating available observations from all randomised participants is a further limitation, as its influence on the longitudinal estimates could not be assessed. Available-data modelling within this selected cohort cannot represent children who discontinued treatment or establish tolerability in all starters.

Several limitations should be acknowledged. All participants received atropine, and no untreated, vehicle-controlled, or preservative-free comparator was included. Baseline measurements therefore constituted a temporal reference rather than a concurrent control, and the observed longitudinal changes cannot be causally attributed to atropine or interpreted as demonstrating equivalence to no treatment. As a secondary analysis, the study was not prospectively powered for the ocular surface outcomes and may have had limited ability to detect very small changes, uncommon adverse events, or subgroup interactions. Accordingly, the absence of statistically significant differences should not be interpreted as definitive evidence of an absence of harm. Ocular surface characterisation was also limited to fNIBUT and OSDI and did not include staining, tear-volume or osmolarity measurements, lipid-layer assessment, meibography, or detailed examination of the eyelids and meibomian glands. ECD provided complementary information on corneal endothelial cell density but is not a direct measure of ocular surface health. Only one technically acceptable fNIBUT recording was retained per assessment. Repeating inadequate attempts did not permit assessment of within-session repeatability or reduce variability by averaging valid readings, potentially reducing precision and power. Increasing age and familiarity may have facilitated ECD acquisition at later visits, but this was not formally assessed.

OSDI was developed for adults, and validity in very young children is uncertain. Unrecorded parental assistance may represent proxy reporting and precluded an unassisted-questionnaire analysis. Follow-up missingness was concentrated in SV, with no established explanation for the imbalance. The circumstances of acquisition failure or questionnaire non-completion do not establish MAR, and the overall OSDI sensitivity analysis did not resolve the DIMS–SV comparison or completer-selection bias. Within-family multiplicity adjustment was used, but the large number of exploratory analyses still warrants caution. Screen exposure was self-reported, adherence was assessed indirectly, environmental conditions were unrecorded, and individual instillation-to-examination intervals were unavailable. The single-centre setting and incomplete formulation characterisation limit generalisability.

Morning assessment does not exclude residual pharmacological effects. The larger pupils at 24 months are compatible with persistent mydriasis, but lack of an untreated comparator and individual timing data limits attribution. Pupil diameter neither quantifies residual cycloplegia nor demonstrates an effect on blinking or tear-film stability. Potential atropine and preservative effects on these measurements could not be separated. Although adverse events were elicited, separate counts for all ocular symptoms were not available for this secondary analysis.

## 5. Conclusions

Among children completing 24 months of the ASPECT trial, no deterioration was detected in measured tear-film stability, ocular symptoms, or endothelial cell density. OSDI scores decreased in the primary model, but the statistical evidence was sensitive to missing-data assumptions and age restriction. Selection of completers, differential missingness, unrecorded parental assistance, incomplete formulation characterisation, and absence of a concurrent untreated comparator preclude firm conclusions about long-term ocular surface tolerability. These observations should not be generalised to all children starting compounded 0.025% atropine.

## Figures and Tables

**Table 1:** Baseline characteristics of participants.

Variables	Available, *n*	Overall Cohort (*N* = 101)
Participants, *n*	101	101
Sex, *n* (%)	101	Males: 54 (53.5) Females: 47 (46.5)
Age, years	101	10.02 ± 2.45
SFChT, µm	94	293.16 ± 62.76
fNIBUT, s	98	14.41 ± 7.91
OSDI score	101	14.73 ± 16.10
ECD, cells/mm^2^	90	3015.32 ± 316.95
SER, D	96	−2.26 ± 1.19
AL, mm	96	24.40 ± 0.88
Spectacle group, *n* (%)	101	SV: 48 (47.5) DIMS: 53 (52.5)
Age group, *n* (%)	101	<8 years: 16 (15.8) ≥8 years: 85 (84.2)
Screen use at school, *n* (%)	73	No: 45 (61.6) Yes: 28 (38.4)
Weekday screen exposure, *n* (%)	73	<2 h: 45 (61.6) 2–4 h: 15 (20.5) >4 h: 13 (17.8)
Weekend screen exposure, *n* (%)	72	<2 h: 26 (36.1) 2–4 h: 23 (31.9) >4 h: 23 (31.9)
Parent-assisted OSDI questionnaires	Not recorded	Not quantifiable retrospectively

Data are mean ± SD or *n* (%). Available *n* is the denominator for each variable; percentages may not sum to 100% because of rounding. All screen responses refer to baseline; the questionnaire was introduced after recruitment began and was not given to the earliest recruits. Missing screen responses were not classified as no exposure. Parental assistance with OSDI was not systematically recorded. SER, spherical equivalent refraction; AL, axial length; SFChT, subfoveal choroidal thickness; fNIBUT, first non-invasive tear-film break-up time; OSDI, Ocular Surface Disease Index; ECD, endothelial cell density; SV, single-vision; DIMS, Defocus Incorporated Multiple Segments.

**Table 2:** Longitudinal changes in fNIBUT and OSDI.

	Estimated Mean (95% CI)			MD (95% CI); *p* Value		
Outcome	Baseline	12 Months	24 Months	12 Months vs. Baseline	24 Months vs. Baseline	24 vs. 12 Months
fNIBUT, s	14.3 (12.7 to 16.0)	14.6 (12.9 to 16.3)	16.9 (15.3 to 18.6)	0.25 (−2.72 to 3.21); 0.868	2.60 (−0.37 to 5.57); 0.103	2.35 (−0.64 to 5.35); 0.138
fNIBUT, *n*	98	94	94			
OSDI score	14.7 (11.9 to 17.6)	11.0 (8.1 to 13.9)	9.6 (6.6 to 12.6)	−3.72 (−8.38 to 0.94); 0.138	−5.15 (−9.93 to −0.36); 0.028	−1.43 (−6.27 to 3.42); 0.868
OSDI, *n*	101	96	88			

Note: Values are model-estimated means with 95% confidence intervals (CIs) derived from linear mixed-effects models. Mean differences were calculated as the later visit minus the earlier visit; therefore, positive values indicate an increase over time and negative values indicate a decrease. For the two primary outcomes of this secondary analysis, the six between-visit comparisons were treated as a single family: *p*-values were adjusted using the Holm method and the corresponding 95% CIs are simultaneous Bonferroni intervals. Per-visit estimated means are reported with unadjusted 95% CIs. Abbreviations: CI, confidence interval; MD, mean difference; fNIBUT, first non-invasive tear-film break-up time; OSDI, Ocular Surface Disease Index.

## Data Availability

The datasets generated and/or analysed during the current study are available from the corresponding author upon reasonable request.

## Abbreviations

The following abbreviations are used in this manuscript:

AL	axial length
ASPECT	Atropine and Spectacle Lens Combination Treatment
BCVA	best-corrected visual acuity
CCT	central corneal thickness
CI	confidence interval
CONSORT	Consolidated Standards of Reporting Trials
D	dioptre
DED	dry eye disease
DIMS	Defocus Incorporated Multiple Segments
ECD	corneal endothelial cell density
ETDRS	Early Treatment Diabetic Retinopathy Study
fNIBUT	first non-invasive tear-film break-up time
GEE	generalised estimating equations
LAMP	Low-Concentration Atropine for Myopia Progression
logMAR	logarithm of the minimum angle of resolution
MAR	missing at random
MD	mean difference
MNAR	missing not at random
OCT	optical coherence tomography
OR	odds ratio
OSDI	Ocular Surface Disease Index
REML	restricted maximum likelihood
SD	standard deviation
SER	spherical equivalent refraction
SFChT	subfoveal choroidal thickness
STROBE	Strengthening the Reporting of Observational Studies in Epidemiology
SV	single-vision
TBUT	tear-film break-up time
TFOS DEWS II	Tear Film & Ocular Surface Society Dry Eye Workshop II

## Appendix A. Additional Analyses

### Appendix A.1. Subfoveal Choroidal Thickness

**Table A1:** Exploratory correlations between fNIBUT and SFChT at each visit.

Visit	*N*	Pearson’s r	95% CI	*p*-Value
Baseline	91	0.044	−0.16 to 0.25	0.680
12 months	90	0.180	−0.03 to 0.37	≈0.09
24 months	83	0.013	−0.20 to 0.23	0.909

*n* is the number with both measurements. The 12-month two-sided, unadjusted Pearson *p*-value was recalculated from the reported rounded r = 0.180 and *n* = 90 using t = r√[(*n* − 2)/(1 − r^2^)], with 88 degrees of freedom, giving *p* approximately 0.09. This is a summary-statistic consistency correction, not a reanalysis of the participant-level pairs. The coefficients, CIs, and remaining *p*-values reproduce the revised statistical output. SFChT, subfoveal choroidal thickness; fNIBUT, first non-invasive tear-film break-up time; CI, confidence interval.

### Appendix A.2. Sex

**Table A2:** Model-estimated differences in fNIBUT and OSDI scores according to sex during the 24-month follow-up.

Outcome	Visit	Estimated Difference (Girls − Boys)	95% CI	*p*-Value
fNIBUT, s	Baseline	−1.51	−5.59 to 2.57	1.000
	12 months	0.12	−3.30 to 3.54	1.000
	24 months	−0.49	−3.91 to 2.92	1.000
OSDI score	Baseline	−3.46	−10.48 to 3.57	0.710
	12 months	1.78	−5.42 to 8.98	1.000
	24 months	−0.66	−6.77 to 5.45	1.000

Differences were calculated as girls minus boys; negative values indicate lower estimated values in girls. The statistical report specifies that estimates and 95% CIs were obtained from sex-by-visit contrasts of longitudinal mixed-effects models, with Holm-adjusted *p*-values and Bonferroni-adjusted 95% CIs over the three visit-specific comparisons within each outcome. CI, confidence interval; fNIBUT, first non-invasive tear-film break-up time; OSDI, Ocular Surface Disease Index.

### Appendix A.3. Age-Restricted OSDI Sensitivity Analysis

**Table A3:** OSDI sensitivity analysis restricted to baseline age ≥ 8 years.

Contrast	MD (Points)	95% CI	Adjusted *p*
12 months − baseline	−3.30	−8.73 to 2.13	0.319
24 months − baseline	−5.40	−10.98 to 0.19	0.064
24 − 12 months	−2.10	−7.77 to 3.57	0.648

The cohort contained 16 children aged <8 years (15.8%) and 85 aged ≥8 years (84.2%). The sensitivity analysis used available OSDI measurements within the latter group. Differences are later minus earlier visit; negative values indicate lower symptoms. The age-restricted analysis adjusted *p*-values using Holm and CIs using Bonferroni over the six fNIBUT/OSDI visit contrasts. An unassisted-questionnaire analysis was not possible because parental assistance was not recorded.

### Appendix A.4. OSDI Missing-Data Sensitivity Analysis

**Table A4:** Sensitivity of the 24-month OSDI difference to missing-data assumptions.

Delta Added to Missing Scores	24 Months − Baseline MD	Bonferroni 95% CI	Bonferroni *p*
0	−5.04	−9.84 to −0.24	0.034
2.5	−4.72	−9.53 to 0.09	0.057
5	−4.40	−9.23 to 0.43	0.095
10	−3.76	−8.65 to 1.13	0.247

A supplementary overall-cohort OSDI sensitivity analysis used 2000 predictive mean matching imputations, with five donors, 20 iterations, and seed 20260928. Predictors were baseline OSDI, the other follow-up OSDI, baseline age, and sex. The sensitivity analysis pooled the cohort and did not include spectacle allocation; it therefore does not assess robustness of the DIMS–SV comparison. After imputation under a missing-at-random (MAR) reference, positive delta shifts were added to originally missing scores at 12 and 24 months and capped at 100. Each completed dataset was analysed using a visit-plus-random-intercept REML model. Results were pooled using Rubin’s rules with Barnard–Rubin degrees of freedom (complete-data degrees of freedom set to 100). A conservative Bonferroni correction for six contrasts was applied to *p*-values and CIs within each scenario, distinct from the primary Holm procedure. Delta values of 0, 2.5, 5, and 10 points illustrate sensitivity to departures from MAR. A supplementary scenario shifted only 24-month missing values (Table A4). Observed OSDI counts were 101, 96, and 88 at baseline, 12 months, and 24 months. Delta represents an assumed worsening of missing scores relative to MAR predictions, not an observed difference. The MAR case uses delta = 0. Shifting only missing 24-month values by 2.5 points gave MD −4.72 (95% CI, −9.53 to 0.09; *p* = 0.057). The analysis does not include the ten excluded participants or establish robustness of the spectacle comparison. The direction of the OSDI decrease persisted in the displayed scenarios, but statistical significance did not.

### Appendix A.5. Endothelial Cell Density

**Table A5:** Endothelial cell density during 24-month follow-up.

Visit	*n*	ECD Cells/mm^2^, Mean ± SD	Mean Difference, Cells/mm^2^	95% CI	*p*-Value
Baseline	90	3015.32 ± 316.95	-	-	-
12 months	95	3055.00 ± 237.56	37.9 *	−18.5 to 94.3	0.319
24 months	98	3045.42 ± 242.02	25.4 *	−30.3 to 81.0	0.544
24 vs. 12 months	-	-	−12.5 †	−67.3 to 42.2	0.581

* Mean difference relative to baseline. † Mean difference between the 24- and 12-month visits. Values at each visit are presented as mean ± standard deviation. Longitudinal comparisons were obtained from the linear mixed-effects model. ECD, endothelial cell density; CI, confidence interval; SD, standard deviation. Pairwise *p*-values are Holm-adjusted and CIs are simultaneous Bonferroni intervals over three contrasts. The 11 unavailable baseline measurements reflected measurement difficulties; subsequent denominators reflect visit-specific availability within the same 101 participants.

### Appendix A.6. Combined Symptom and Tear-Film Criterion

**Table A6:** Exploratory combined symptom and tear-film criterion during follow-up. (**A**) Observed frequency and model-estimated probability; (**B**) Pairwise longitudinal comparisons.

(**A**)				
**Visit**	**DED, *n*/*N* (%)**		**GEE Probability, % (95% CI)**	
Baseline	13/98 (13.3)		13.2 (7.8–21.5)	
12 months	8/93 (8.6)		8.8 (4.5–16.5)	
24 months	5/86 (5.8)		5.9 (2.5–13.3)	
(**B**)				
**Comparison**		**OR**	**95% CI**	***p*** **-Value**
Baseline vs. 12 months		1.58	0.51–4.86	0.657
Baseline vs. 24 months		2.43	0.68–8.64	0.279
12 vs. 24 months		1.54	0.48–4.93	0.657

The combined criterion was OSDI ≥ 13 and fNIBUT < 10 s at the same visit. GEE used a binomial family, logit link, exchangeable working correlation, and robust standard errors. Probabilities are population-averaged, with pointwise 95% CIs. Pairwise ORs compare the first visit with the second; *p*-values are Holm-adjusted and CIs are simultaneous Bonferroni intervals across three contrasts. Sparse events limit precision. CI, confidence interval; OR, odds ratio.

### Appendix A.7. Spectacle Group Comparison

**Table A7:** Model-estimated differences in fNIBUT and OSDI scores according to spectacle type during the 24-month follow-up.

	Visit	*n* (DIMS/SV)	Estimated Difference (DIMS-SV)	95% CI	*p*-Value
fNIBUT, s	Baseline	53/45	−2.56	−6.79 to 1.68	0.441
	12 months	53/41	0.48	−3.80 to 4.76	1.000
	24 months	53/41	−0.36	−4.61 to 3.89	1.000
OSDI score	Baseline	53/48	−4.09	−11.41 to 3.23	0.538
	12 months	53/43	−3.69	−11.08 to 3.70	0.538
	24 months	53/35	−1.15	−8.89 to 6.58	0.720

Differences are DIMS minus SV. Pairwise *p*-values are Holm-adjusted and CIs are simultaneous Bonferroni intervals across three visits within each outcome. *n* denotes available DIMS/SV observations. Follow-up missingness was concentrated in SV, limiting interpretation.

### Appendix A.8. Age

**Table A8:** Effect of baseline age on fNIBUT and OSDI trajectories in longitudinal mixed-effects models.

Outcome	Effect	Coefficient (95% CI)	*p*-Value
fNIBUT	Baseline age	0.56 (−0.13 to 1.25)	0.112
	Age × 12 months vs. baseline	−0.39 (−1.30 to 0.51)	0.395
	Age × 24 months vs. baseline	−0.36 (−1.26 to 0.54)	0.435
	Age × time (global)		0.640
OSDI	Baseline age	0.18 (−0.98 to 1.35)	0.758
	Age × 12 months vs. baseline	0.85 (−0.55 to 2.25)	0.233
	Age × 24 months vs. baseline	0.18 (−1.24 to 1.61)	0.803
	Age × time (global)		0.458

Baseline age was continuous; baseline was the reference visit. Interaction coefficients represent differences in age slopes relative to baseline. OSDI results were independently recalculated using a REML random-intercept model (101 participants; 285 observations). Coefficient *p*-values and 95% CIs use unadjusted normal Wald inference; the global interaction uses a two-degree-of-freedom Wald chi-square test. fNIBUT results reproduce the revised statistical output. fNIBUT, first non-invasive tear-film break-up time; OSDI, Ocular Surface Disease Index.

### Appendix A.9. Screen Exposure

**Table A9:** Pairwise comparisons of fNIBUT and OSDI scores according to reported digital screen exposure.

Visit	Comparison	fNIBUT, MD (95% CI)	*p* Value	OSDI, MD (95% CI)	*p* Value
Screen use at school (yes vs. no)					
Baseline	Yes vs. no	−0.31 (−4.12 to 3.50)	0.874	1.00 (−5.40 to 7.40)	0.759
12 months	Yes vs. no	3.48 (−0.43 to 7.39)	0.081	−0.24 (−6.90 to 6.41)	0.943
24 months	Yes vs. no	−0.18 (−4.10 to 3.74)	0.928	−0.93 (−7.78 to 5.93)	0.790
Weekday screen exposure					
Baseline	2–4 vs. <2 h	−1.51 (−7.01 to 4.00)	1.000	9.41 (0.39 to 18.44)	**0.038**
	>4 vs. <2 h	1.34 (−4.88 to 7.57)	1.000	5.19 (−5.23 to 15.62)	0.460
	>4 vs. 2–4 h	2.85 (−4.40 to 10.10)	1.000	−4.22 (−16.27 to 7.84)	0.460
12 months	2–4 vs. <2 h	−1.89 (−7.34 to 3.56)	0.405	4.12 (−5.15 to 13.38)	0.677
	>4 vs. <2 h	5.43 (−1.04 to 11.90)	0.088	5.29 (−5.22 to 15.79)	0.677
	>4 vs. 2–4 h	7.31 (−0.03 to 14.66)	0.051	1.17 (−10.99 to 13.34)	0.816
24 months	2–4 vs. <2 h	3.19 (−2.71 to 9.09)	0.480	−0.24 (−10.11 to 9.63)	0.953
	>4 vs. <2 h	−1.22 (−7.46 to 5.03)	0.639	6.10 (−4.75 to 16.95)	0.528
	>4 vs. 2–4 h	−4.41 (−11.95 to 3.14)	0.480	6.34 (−6.47 to 19.45)	0.528
Weekend screen exposure					
Baseline	2–4 vs. <2 h	−0.56 (−6.14 to 5.03)	1.000	3.70 (−5.32 to 12.71)	0.386
	>4 vs. <2 h	0.54 (−4.94 to 6.01)	1.000	8.62 (−0.40 to 17.63)	0.066
	>4 vs. 2–4 h	1.09 (−4.49 to 6.68)	1.000	4.92 (−4.18 to 14.02)	0.386
12 months	2–4 vs. <2 h	−2.67 (−8.30 to 2.97)	0.510	−4.00 (−13.32 to 5.32)	0.903
	>4 vs. <2 h	0.98 (−4.66 to 6.61)	0.676	−1.05 (−10.21 to 8.11)	0.903
	>4 vs. 2–4 h	3.64 (−2.05 to 9.33)	0.372	2.95 (−6.46 to 12.35)	0.903
24 months	2–4 vs. <2 h	3.23 (−2.36 to 8.83)	0.494	−3.08 (−12.54 to 6.38)	0.433
	>4 vs. <2 h	1.89 (−3.78 to 7.55)	0.845	6.37 (−3.45 to 16.19)	0.238
	>4 vs. 2–4 h	−1.35 (−7.22 to 4.52)	0.845	9.45 (−0.31 to 19.21)	0.061

Differences are first minus second category, using baseline exposure. Weekday/weekend contrasts use Holm *p*-values and simultaneous Bonferroni 95% CIs across three comparisons per visit and outcome; school use has one unadjusted comparison. Bold: *p* < 0.05. MD, mean difference; CI, confidence interval; fNIBUT, first non-invasive tear-film break-up time; OSDI, Ocular Surface Disease Index.

### Appendix A.10. Reported Adverse Events and Treatment Discontinuations

**Table A10:** Documented adverse events and treatment discontinuations in the parent ASPECT trial.

Outcome	SV, *n*	DIMS, *n*
Early atropine discontinuation associated with refusal of instillation ^1^	1	0
Photophobia requiring photochromic DIMS lenses at 6 months ^2^	0	2
Additional treatment-related discontinuation during months 12–24	0	0
Serious ocular adverse events reported	0	0
Serious systemic adverse events reported	0	0

Counts reproduce the available 24-month safety summary and include events among participants who withdrew; they are not restricted to the 101-completer outcome-analysis cohort. Percentages are not calculated because a verified event-specific safety denominator was unavailable. These rows are not a complete accounting of withdrawals or symptom incidence. ^1^ The SV participant complained about instillation and refused further administration; the parent stopped the drops after approximately two days. No specific ocular surface adverse manifestation was documented. This participant is included among the three SV drop-administration withdrawals in Figure 1. Refusal may reflect discomfort or poor acceptability and is not proof of absence of a tolerability problem. The other administration difficulties are described in participant flow. ^2^ Both participants remained in follow-up. Separate counts for all stinging, hyperaemia, allergic conjunctivitis, and near-vision blurring were unavailable. An unlisted event should not be interpreted as verified zero incidence. SV, single-vision; DIMS, Defocus Incorporated Multiple Segments.
